# Understanding Enhanced Melt Memory in Poly(Octamethylene Carbonate)-Based Random Copolycarbonates with Mixed Isodimorphic/Isomorphic Crystallization

**DOI:** 10.1021/acs.macromol.5c02607

**Published:** 2026-01-01

**Authors:** Yilong Liao, Ricardo A. Pérez-Camargo, Jon Maiz, Alejandro J. Müller

**Affiliations:** 1 School of Materials Science and Engineering, and State Key Laboratory of High Performance Roll Materials and Composite Forming, 12605 Tianjin University, Tianjin 300072, P.R. China; 2 State Key Laboratory of Advanced Polymer Materials, Sichuan University, Chengdu 610065, P.R. China; 3 POLYMAT and Department of Polymers and Advanced Materials: Physics, Chemistry and Technology, Faculty of Chemistry, University of the Basque Country UPV/EHU, Donostia-San Sebastián 20018, Spain; 4 Centro de Física de Materiales (CFM-MPC), CSIC-UPV/EHU, Paseo Manuel de Lardizabal 5, Donostia-San Sebastian 20018, Spain; 5 Ikerbasque, Basque Foundation for Science, Plaza Euskadi 5, Bilbao 48009, Spain

## Abstract

Poly­(hexamethylene carbonate-*ran*-octamethylene carbonate) (PC6/PC8), poly­(heptamethylene carbonate-*ran*-octamethylene carbonate) (PC7/PC8), and poly­(dodecamethylene-*ran*-octamethylene carbonate) (PC12/PC8) are the first random copolymers known to crystallize with a mixed isodimorphic and isomorphic mode. Here, we investigate for the first time how this dual crystallization mode affects melt memory. Self-nucleation experiments, combined with synchrotron X-ray scattering, polarized light microscopy, and dielectric spectroscopy, show that crystallization in the isodimorphic mode decreases melt memory as the comonomer content increases, as expected. Conversely, compositions that crystallize in the isomorphic mode, where a new crystalline phase forms that is different from the parent components or their polymorphs, display increased melt memory, with *Domain IIa* widths—directly related to melt memory—reaching up to 30 °C, significantly surpassing those in homopolymers. This heightened melt memory is due to stronger intermolecular interactions within the new phase, as shown by higher dielectric constants. These results establish the crucial role of intermolecular interactions within the crystal lattice in determining enhanced melt memory in copolymers showing both isodimorphic and isomorphic crystallization, highlighting the critical role of comonomer incorporation in tuning crystallization behavior and melt memory.

## Graphical abstract

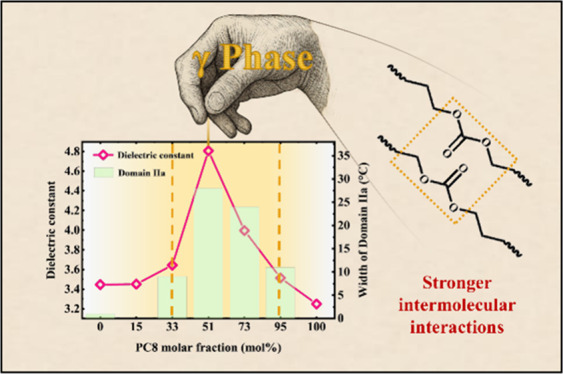

## 1 Introduction

Random copolymerization is a common strategy used to tailor polymer properties because it allows combining the characteristics of Polymer A (P_A_) and Polymer B (P_B_) without issues of miscibility in the melt state, thanks to covalent bonds between the repeating units. During crystallization, the behavior of a P_A_/P_B_ random copolymer depends on whether A-units are fully or partially incorporated into P_B_ crystals or are completely excluded to the amorphous phase, and *vice versa*. This leads to three well-known crystallization modes: *comonomer exclusion* (complete exclusion), *isomorphism* (complete inclusion), and *isodimorphism*, which represents a balance between exclusion and inclusion.^1–6^

Isodimorphic random copolymers have recently attracted significant attention because they can crystallize over the entire composition range without requiring the strict structural conditions needed for cocrystal formation in isomorphic systems.^1–6^ This makes them ideal for tuning material properties, such as thermal transitions, degree of crystallinity, and mechanical properties, simply by altering the composition. Several criteria have been established to identify this behavior: crystallization across the whole composition range, unit cell distortions that permit comonomer inclusion,^1,2^ and a pseudoeutectic behavior in first-order thermal transitions, degree of crystallinity (*X* *_c_*), and mechanical properties that depend on *X* *_c_*. Despite considerable progress, recent studies have revealed complex systems that display features of isodimorphism but also show characteristics of other modes, such as *isodimorphism/isomorphism* or *isodimorphism/comonomer exclusion*, indicating the presence of mixed crystallization modes.

In our recent studies, we identified a mixed mode, specifically isodimorphism/isomorphism, in random copolycarbonates.^4,5^ They display typical isodimorphic features, including crystallization throughout the entire composition range, pseudoeutectic melting behavior, and parent-like unit cells with lattice distortions caused by partial comonomer inclusion. At intermediate compositions, however, isomorphic traits appear, with new crystalline phases distinct from P_A_ or P_B_ and linear changes in properties such as *d*-spacing and melting points. Although the emergence of this new crystalline phase (the γ phase) is clearly evident, its detailed crystal structure has not yet been determined and is currently inferred from diffraction and spectroscopic evidence obtained in relatively low molecular weight samples, which cannot be highly oriented to produce fibers due to their fragile nature. These mixed-phase compositions show unexpected *X* *_c_* values, either exceeding those of the homopolymers or deviating from pseudoeutectic trends.

Knowledge about this mixed isodimorphic/isomorphic mode remains limited. One key phenomenon that remains underexplored is how melt memory can be affected by this peculiar mixed crystallization mode. Generally, heating a polymer above its melting range (25–30 °C) erases thermal history, resulting in an isotropic melt. Upon cooling, crystallization is governed by external heterogeneities (e.g., catalyst residues). However, when melting is incomplete or occurs within the melting range, residual order can remain, which can reduce the nucleation energy barrier for subsequent crystallization and accelerate crystallization, a process known as self-nucleation.^7–11^ When this nucleation occurs above the full melting range, the acceleration is attributed to melt memory, a case characterized by the total melting of the polymer just above the end of its melting range, where no crystal fragments that could act as self-seeds remain, yet the polymer chains somewhat ″remember″ their conformations in the crystalline state.^9^

Although the exact origin of melt memory is still uncertain, several hypotheses exist: presence of ordered chain segments,^12–14^ aggregations of chain sequences,^15,16^ and intermolecular interactions.^17–20^ The complexity of this effect is such that it shows specific dependence on the material’s nature. In nonpolar polymers such as polyethylene (PE) or isotactic polypropylene (iPP), melt memory is absent, as only weak intermolecular interactions (van der Waals forces) are present between the chains, and self-nucleation is entirely due to unmolten crystal fragments that can act as self-seeds.^9,14,15^ However, incorporating even small amounts of comonomers in polyolefins can induce strong melt memory, even above equilibrium melting temperatures (*T* *_m_* *°*).^10,11,15,21–24^ Alamo et al.^15^ attributed this to persistent crystallizable sequences segregating in the melt, while simulations by Hu et al.^25^ suggested that amorphous-phase enrichment around former crystals alters local thermodynamics, promoting nucleation. Although the crystal loses its three-dimensional order upon melting, forming a relaxed melt, the enrichment of crystallizable segments in the initial crystalline regions alters the thermodynamic properties of the local melt, reducing the nucleation barrier for subsequent recrystallization. Interestingly, even in cases where comonomers are included in the lattice, as in iPP with pentene counits or stereodefects, melt memory can persist due to structural irregularities impeding complete melt homogenization.^26–28^ This behavior is explained by the fact that although the comonomers can be present within the lattice, they still act as defects, increasing the difficulty of sequence diffusion and homogenization to some extent.

In contrast, polar polymers (e.g., polyesters, polycarbonates, polyamides) display stronger melt memory because of strong intermolecular interactions (hydrogen bonding, dipole interactions). Even at temperatures above *T* *_m_* *°*, the complete relaxation of polyamide 6 (PA6) melt takes a very long time, which is undoubtedly due to the strong intermolecular hydrogen bonding.^29^ Through thermo-rheology, the melt memory observed in polycaprolactone (PCL) has been attributed to dipole–dipole interactions, providing additional entanglements that hinder molecular chain diffusion. It is important to note that these intersegmental interactions differ from conventional topological entanglements, as they gradually relax with increasing temperature.^30^ Sangroniz et al. showed that melt memory strength increases with higher polarity (fewer methylene units), reinforcing the role of polar interactions.^19^

However, in polar copolymers, the situation is quite different from what occurs in nonpolar copolymers, such as polyolefins. For example, in an isodimorphic copolymer like poly­[(butylene succinate)-*ran*-(ε-caprolactone)] (PBS/PCL), researchers have discovered that adding even a small amount of comonomer (as little as 1 mol %) can significantly reduce melt memory.^31^ This effect is even more pronounced when the added comonomer units (in this case, butylene succinate) are mostly left out of the crystalline structure formed by the main component (caprolactone). The likely explanation is that randomly incorporating these comonomer units breaks the regular packing and intermolecular interactions within the crystals, making it harder for the material to ″remember″ how to crystallize after melting.

Research on melt memory in isodimorphic systems is limited to a few specific cases, so there is still no comprehensive understanding of how including or excluding comonomeric units in the crystal structure influences melt memory. To date, loss of melt memory appears to be a common feature of isodimorphic crystallization. But what about isomorphic crystallization? In these systems, comonomers are fully incorporated into a new single crystalline phase (i.e., the two repeating units that are randomly distributed along the chains can cocrystallize), which raises new questions about the persistence of melt memory. It is precisely this research gap that has motivated this work.

In this study, we have examined how the mixed isodimorphic/isomorphic crystallization mode affects melt memory in three copolycarbonate series: poly­(hexamethylene carbonate-*ran*-octamethylene carbonate) (PC6/PC8), poly­(heptamethylene carbonate-*ran*-octamethylene carbonate) (PC7/PC8), and poly­(dodecamethylene carbonate-*ran*-octamethylene carbonate) (PC12/PC8), especially when the copolymers crystallize in an isomorphic manner. Self-nucleation experiments using differential scanning calorimetry (DSC) showed a significant increase in melt memory at compositions with extensive or total comonomer inclusion. Additional analyses, including synchrotron X-ray scattering and dielectric measurements, indicate that this increase is due to enhanced or restored intermolecular interactions when comonomer inclusion is prominent. This research uncovers a new aspect of the mixed isodimorphic/isomorphic crystallization mode and provides valuable insights into the melt memory of polar copolymers.

## 2 Experimental Part

### 2.1 Materials

The PC8-based copolymers investigated in this study, including PC6/PC8, PC7/PC8, and PC12/PC8, were synthesized and characterized in our previous works.^4,5^ The synthetic procedures and chemical structures are illustrated in Scheme 1. Detailed microstructural features and compositional information were determined via ^1^H and ^13^C NMR spectroscopy, as described in the Supporting Information (Figures S1–S3).

**Scheme 1:**
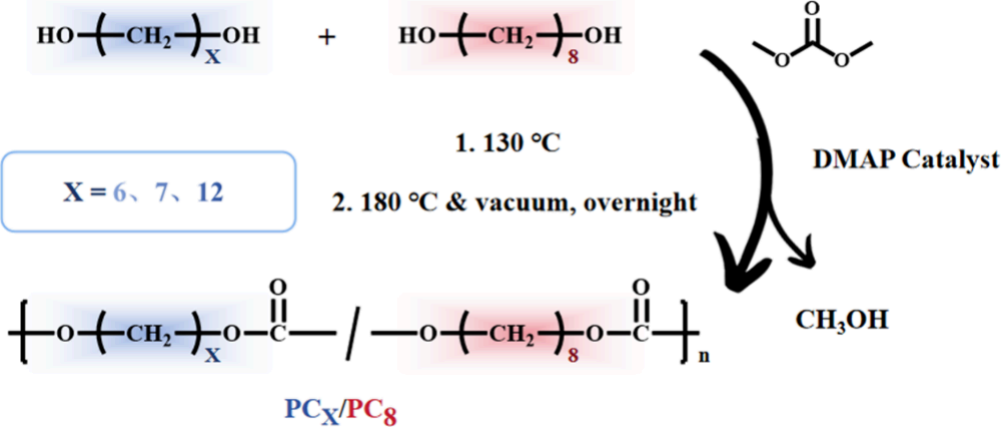
Illustration of Aliphatic Copolycarbonates Synthesized via Polycondensation of Diols with Dimethyl Carbonate

### 2.2 Differential Scanning Calorimetry (DSC)

A PerkinElmer 8000 calorimeter connected to an Intracooler II cooling system was used to investigate the self-nucleation behavior of PC8-based random copolycarbonates. For precise instrument calibration, high-purity indium and tin standards were employed. Approximately 5 mg of each sample was sealed in aluminum DSC pans and placed into the DSC apparatus. All thermal scans were conducted at a constant heating/cooling rate of 20 °C/min under an ultrapure nitrogen atmosphere (flow rate: 20 mL/min). Self-nucleation (SN) experiments were performed following a similar thermal protocol to that established by Fillon et al.^8^ and reviewed by Müller et al.,^9,14^ as shown in Scheme 2. Initially, the sample was heated to 100 °C and held for 3 min to erase prior thermal history and achieve a fully isotropic melt. The melt was then cooled to −40 °C to obtain a standard semicrystalline state (i.e., the sample crystallizes from the isotropic melt during cooling at a constant rate of 20 °C/min) and held for 1 min at this low temperature. Subsequently, the standard crystals were heated up to a predetermined self-nucleation temperature (*T* *_s_*) and held for 5 min, and then cooled again to −40 °C. A final heating step was applied to melt the sample fully. These steps correspond to the steps (1) to (5) in Scheme 2. Crystallization and melting events during the cooling and subsequent heating after the holding step at *T* *_s_* (see colored lines of Steps 4 and 5 in Scheme 2) were recorded to analyze the self-nucleation behavior of the sample.

**Scheme 2:**
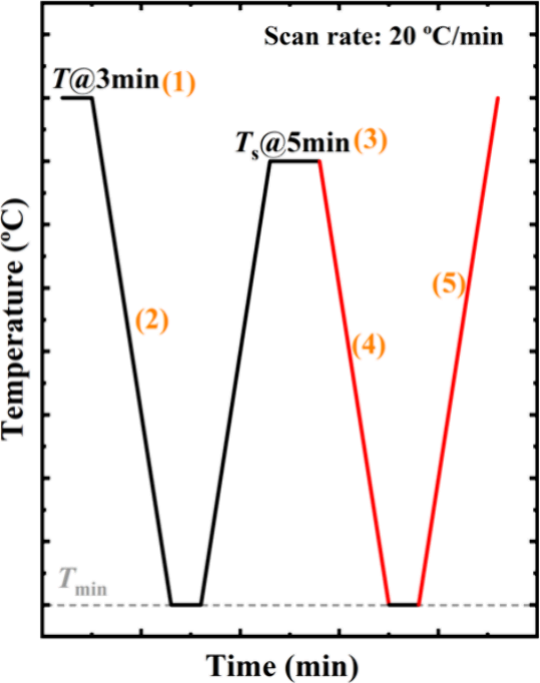
Schematic Representations of the Employed Self-Nucleation Protocol

With this procedure and employing multiple *T* *_s_* temperatures, three self-nucleation *Domains* could be defined by inspecting the DSC scans during cooling and subsequent heating scans.^8,9,14^

#### *Domain I* or Isotropic Melting *Domain*

The polymer sample is in *Domain I* when *T* *_s_* is high enough to completely melt the polymer and produce an isotropic melt where only high-temperature resistant heterogeneities remain. This usually happens at temperatures well above the melting peak of the polymer.

#### *Domain II* or Self-Nucleation *Domain*

Thermal conditioning in this *Domain* causes self-nucleation of the polymer (either by melt memory or by self-seeding produced by unmolten crystal fragments), and as a consequence, the crystallization temperature (*T* *_c_*) is shifted to higher temperatures, as it is a direct function of the nucleation density. Meanwhile, the subsequent melting trace does not reveal any traces of annealing. Müller et al.^9,14^ proposed that *Domain II* should be divided into two *sub-Domains* based on the end of the melting endotherm.

***Domain IIa*** **(** ***DIIa*** **) or** ***Melt Memory Domain*** at higher temperatures within *Domain II*, encompasses temperatures higher than the end of the calorimetric melting (where no latent enthalpy of melting can be detected after the melting peak has reached the liquid baseline of the measurement), in which only melt memory can cause self-nucleation because there are no crystal fragments left in the melt.

***Domain IIb*** **(** ***DIIb*** **) or** ***Self-Seeding Domain***, on the other hand, is located in the lower temperature region of *Domain II*, where the material is not completely molten and the *T* *_s_* temperatures are low enough to leave small crystal fragments (even though they are present, they do not experience annealing during the 5 min at *T* *_s_*).

#### *Domain III* or Self-Nucleation and Annealing *Domain*

If the *T* *_s_* value is lower than a specific temperature within the polymer melting range, it will only produce partial melting. In this case, the sample will be self-seeded and, additionally, the crystals that did not melt will be annealed, and the polymer will be in *Domain III* (*DIII*).

### 2.3 Broadband Dielectric Spectroscopy (BDS)

Dielectric measurements were performed using a Novocontrol Alpha high-resolution analyzer with an applied AC voltage of 1.0 V. Both isothermal (frequency sweep) and isochronal (temperature sweep) spectra were recorded to determine the dielectric properties. Frequency scans were conducted over a range of 10^–1^ to 10^7^ Hz at temperatures from −120 to 25 °C, while temperature sweeps were collected from −150 to 80 °C at selected fixed frequencies between 1 and 10^7^ Hz. For sample preparation, a specific amount of polymer powder was hot-pressed in a MeltPrep equipment to form bubble-free films. These films were then mounted between two gold-plated electrodes of different diameters (20 mm and 30 mm), with the larger-diameter electrode serving as the bottom contact in the sandwich configuration. The film thickness was initially estimated from the difference in electrode thickness before and after pressing. This value was later corrected by measuring the actual thickness of the film after it was peeled off from the electrodes following dielectric measurements. Before measurement, all samples were dried under vacuum at 40 °C overnight while held between the electrodes, and subsequently stored in a desiccator to minimize moisture uptake. The dielectric constant values (ε*’*) were calculated using eq 1:^32^

$$\varepsilon -=\frac{C_{p}d}{\varepsilon_{O}\pi r^{2}}$$ 1

where *C* *_p_* is the real part of the measured capacitance in the parallel configuration, *d* is the film thickness, ε*_o_* is the vacuum permittivity, and *r* is the radius of the top electrode.

### 2.4 Polarized Light Optical Microscopy (PLOM)

The semicrystalline morphology of the samples was examined using an Olympus BX51 polarized optical microscope equipped with a λ plate oriented at 45° to the polarization direction. The thermal protocol was precisely controlled with a THMS600 Linkam hot stage connected to a liquid nitrogen cooling system. For microscopic observation, the samples were placed on a clean glass slide, covered with a thin glass coverslip, and subjected to melt crystallization. The thermal treatment followed the same protocol used in the self-nucleation DSC experiments. After cooling the self-nucleated melt from a selected *T_s_* within the melt memory *Domain* to −40 °C, the resulting morphology was imaged using an Olympus SC50 digital camera.

### 2.5 Wide-Angle X-ray Scattering (WAXS) Experiments

*In-situ* WAXS experiments were performed at the ALBA synchrotron in Barcelona (Spain) using the SN protocol described above for selected *T* *_s_*. WAXS patterns were recorded during the cooling and heating scans after 5 min at each *T* *_s_*. The X-ray source operated at an energy of 12.4 keV (λ = 1.0 Å). In the WAXS configuration, a Rayonix LX255-HS detector with an active area of 230.4 × 76.8 mm^2^ and a pixel size of 44 × 44 μm^2^ was used to record the scattering profiles. The sample-to-detector distance was 15.5 mm, with a tilt angle of 27.3°. WAXS profiles were collected at five-degree intervals. The intensity profile is presented as the scattering intensity plotted against the scattering vector, *q* = 4π sin θλ^–1^, where λ represents the X-ray wavelength and 2θ denotes the scattering angle. Calibration of the scattering vector was performed using chromium­(III).

## 3 Results and Discussion

### 3.1 Understanding the Mixed Isodimorphic/Isomorphic Crystallization Mode

In order to improve the readability, the mixed isodimorphic/isomorphic crystallization mode, introduced in the Introduction and described in previous works,^4,5^ is summarized in Figure 1. Systematic characterization of PC6/PC8, PC7/PC8, and PC12/PC8 highlights the key features of this crystallization mode:^4,5^ (a) crystallization occurs across the whole composition range, with *T* *_c_* and *T* *_m_* following an apparent pseudoeutectic trend (Figure 1a); (b) a new crystalline phase forms (namely γ phase), distinct from that of the parent components or their polymorphs; although its detailed crystal structure has not yet been solved, it is currently identified based on its diffraction and spectroscopic signatures, and its properties vary linearly with composition (Figures 1a and 1b), reflecting isomorphic behavior; (c) the *X* *_c_* deviates from the expected pseudoeutectic behavior typical of purely isodimorphic systems (Figure 1c). The corresponding experimental details are provided in Supporting Information (Figures S4–S6).

**Figure 1:**
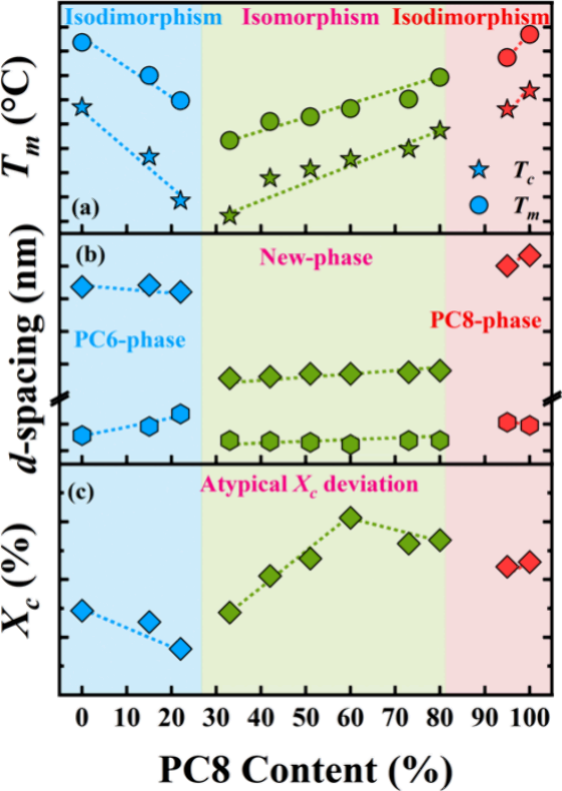
(a) Thermal transition temperatures, (b) *d*-spacings, and (c) crystallinities (*X*_c_) as a function of PC8 content for PC6/PC8 copolymers. Data were extracted from the previous literature.^4^

The behavior shown in Figure 1 is consistent across all PC8-based random copolycarbonates, i.e., PC6/PC8, PC7/PC8, and PC12/PC8. As chain length increases (PC6→PC7→PC12), the composition range forming the new γ phase narrows, and the apparent pseudoeutectic point shifts, possibly because of the comonomer characteristics (even vs odd units).^4,5^ Further study is needed to clarify this effect. In this work, we investigate the melt memory behavior of these mixed mode random copolycarbonates.

### 3.2 Influence of Comonomers on the Self-Nucleation (SN) Effects

Considering the particularities of the mixed isodimorphic/isomorphic crystallization mode, we carried out SN experiments on the copolymers studied here. SN allows us to examine how the different degrees of comonomer inclusion (partial inclusion in compositions that behave in an isodimorphic way and total inclusion for those compositions behaving in an isomorphic manner) influence melt memory.

Figure 2 presents the DSC cooling and subsequent heating scans after the self-nucleation step at the indicated *T*_s_ for selected compositions of the PC6/PC8 copolymer system, taken here as an example. These compositions represent isodimorphic crystallization in the PC6-rich (78/22) and PC8-rich (5/95) regions, as well as isomorphic crystallization at the intermediate composition (40/60). Based on the crystallization and melting behavior observed during these scans, three self-nucleation *Domains* were identified, following the classification explained in the Introduction. To aid visualization, we employed the color code previously used in other works:^9,14^ *Domain I* (red), *Domain II* (blue), and *Domain III* (green). As an example, we briefly describe below the identification criteria for the 78/22 PC6/PC8 copolymer. Further details are provided in Section S3 and References.^9,14^

**Figure 2:**
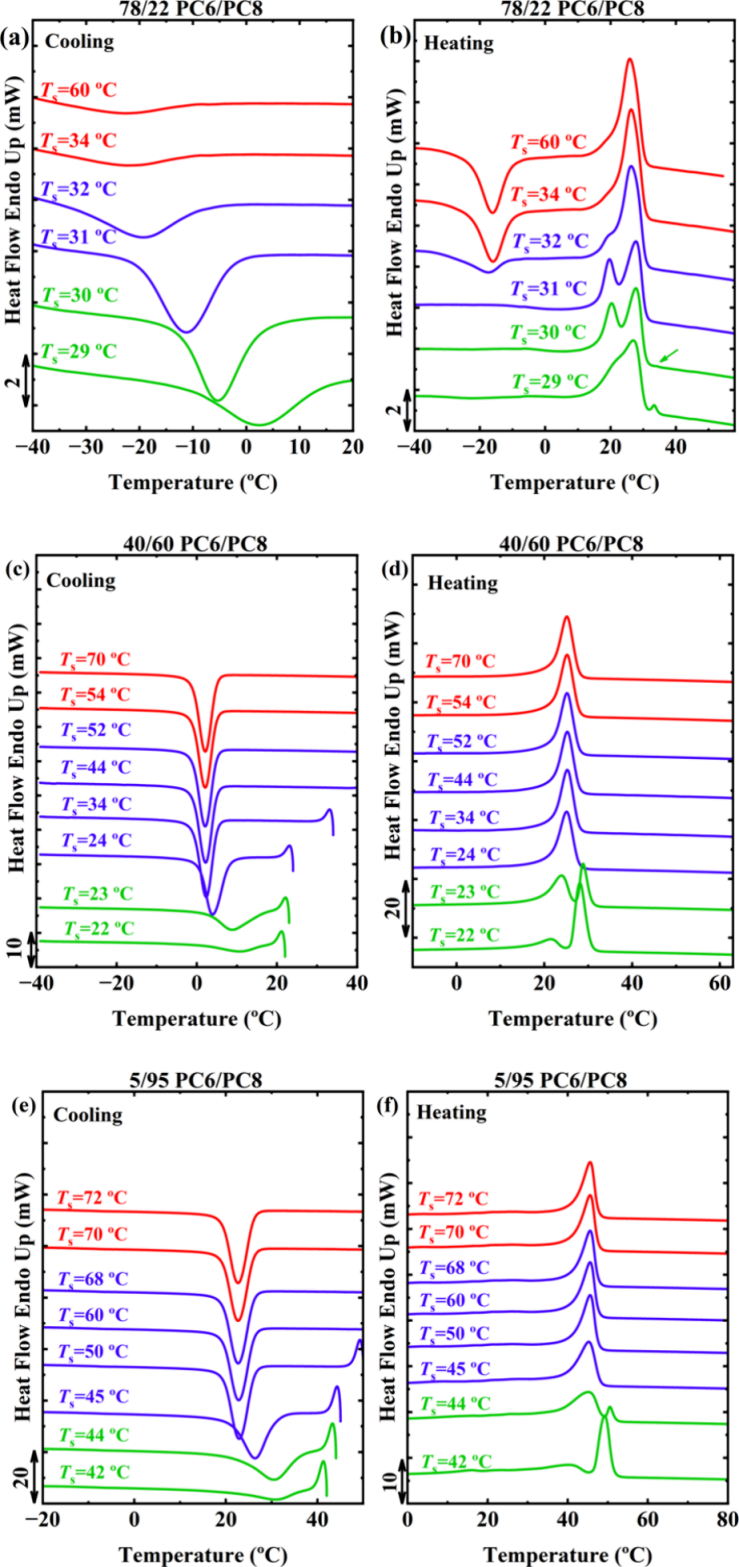
DSC cooling and heating scans after self-nucleation at the indicated temperatures (*T* *_s_*) for (a, b) 78/22, (c, d) 40/60, and (e, f) 5/95 PC6/PC8 copolymers.

For the 78/22 PC6/PC8 copolymer, when *T* *_s_* is equal to or higher than 34 °C, the sample crystallizes upon cooling at the same temperature of −22.9 °C, without modifications in the subsequent melting behavior. Thus, in this *T* *_s_* range, all melt memory is completely erased, resulting in an isotropic melt. This region corresponds to *Domain I* (*DI*), where the *T* *_c_* is highly reproducible and independent of the specific *T* *_s_*.

At *T* *_s_* between 33 and 31 °C, the cooling scans reveal a noticeable increase in *T* *_c_* relative to the standard value observed in *DI*, as shown by the curves at *T* *_s_* = 32 and 31 °C. This *T* *_c_* increase does not alter the subsequent melting behavior and therefore only affects the nucleation density of the samples, i.e., self-nucleation, as evidenced by the PLOM measurements in Figures S7 and S8. To further validate the transition from *Domain I* to *Domain II*, PLOM analysis at the same *T* *_s_* was performed, and nucleation density changes were quantified via Fast Fourier Transform analysis (Figure S9). The results in Figure S10 reveal slight differences in nucleation density between *DI* and *DII*, further confirming the *DII* temperature range identified by DSC.

Once *T* *_s_* falls below 30 °C, the polymer is only partially molten. The unmelted crystals can undergo annealing during the self-nucleation step. Hence, the materials are in the self-nucleation and annealing *Domain*, or *Domain III* (*DIII*). In *Domain III*, *T* *_c_* during cooling typically continues to increase, while in the subsequent heating, a characteristic feature is the appearance of a peak or shoulder at the high-temperature side of the melting endotherm. This new melting peak is attributed to the fusion of annealed crystals during the 5 min at *T* *_s_*.

The same criteria were applied to the other compositions of the PC6/PC8 copolycarbonates, as well as to the PC7/PC8 and PC12/PC8 copolycarbonates. This leads to the identification of SN *Domains* for all the copolycarbonates. Before discussing the SN limits, it is important to note that heating scans after the holding time at *T* *_s_* exhibit interesting melting behaviors. For instance, within *DII* and *DIII*, the melting endotherm of 78/22 PC6/PC8 gradually exhibits a bimodal character, which may arise from the melting and recrystallization of the PC6 phase, or possibly due to a shift in the proportions between the PC6 and γ phases, which coexist in this composition under self-nucleated conditions, leading to sequential melting. In the case of 20/80 PC6/PC8, the complex melting behavior in *DII* and *DIII* might be attributed to the solid–solid phase transition of the PC8 phase influenced by *T* *_s_*. This phenomenon warrants further investigation in the future.

Figure 3 plots the evolution of *T_c_* obtained from DSC cooling scans as a function of *T_s_*, superimposed on the standard DSC melting endotherm for selected compositions of the PC6/PC8 random copolycarbonate. The results for the other PC6/PC8 compositions and PC7/PC8 and PC12/PC8 copolycarbonates are provided in Figures S11–S13. As pointed out by Müller et al.,^9,11,14^ *DII* should be further subdivided into two sub-*Domains* based on the endset temperature of the melting endotherm. The rationale behind this division is to distinguish whether self-nucleation in *DII* arises from melt memory or self-seeding, as explained in the Introduction. In the high-temperature region, i.e., after the melting endotherm ends, referred to as *Domain IIa* (*DIIa*) or melt memory *Domain*, all crystals are completely molten, and the DSC instrument detects no endothermic signal. Therefore, self-nucleation originates purely from a melt that has a memory of the previous crystalline state, i.e, SN arises from melt memory. In the lower temperature region, i.e., before the material completely melts, termed the self-seeding *Domain* or *Domain IIb* (*DIIb*), some unmolten residual crystals persist and act as potent self-seeds for self-nucleation. In Figure 3 and Figures S11–S13, *Domain II* was divided where possible.

**Figure 3:**
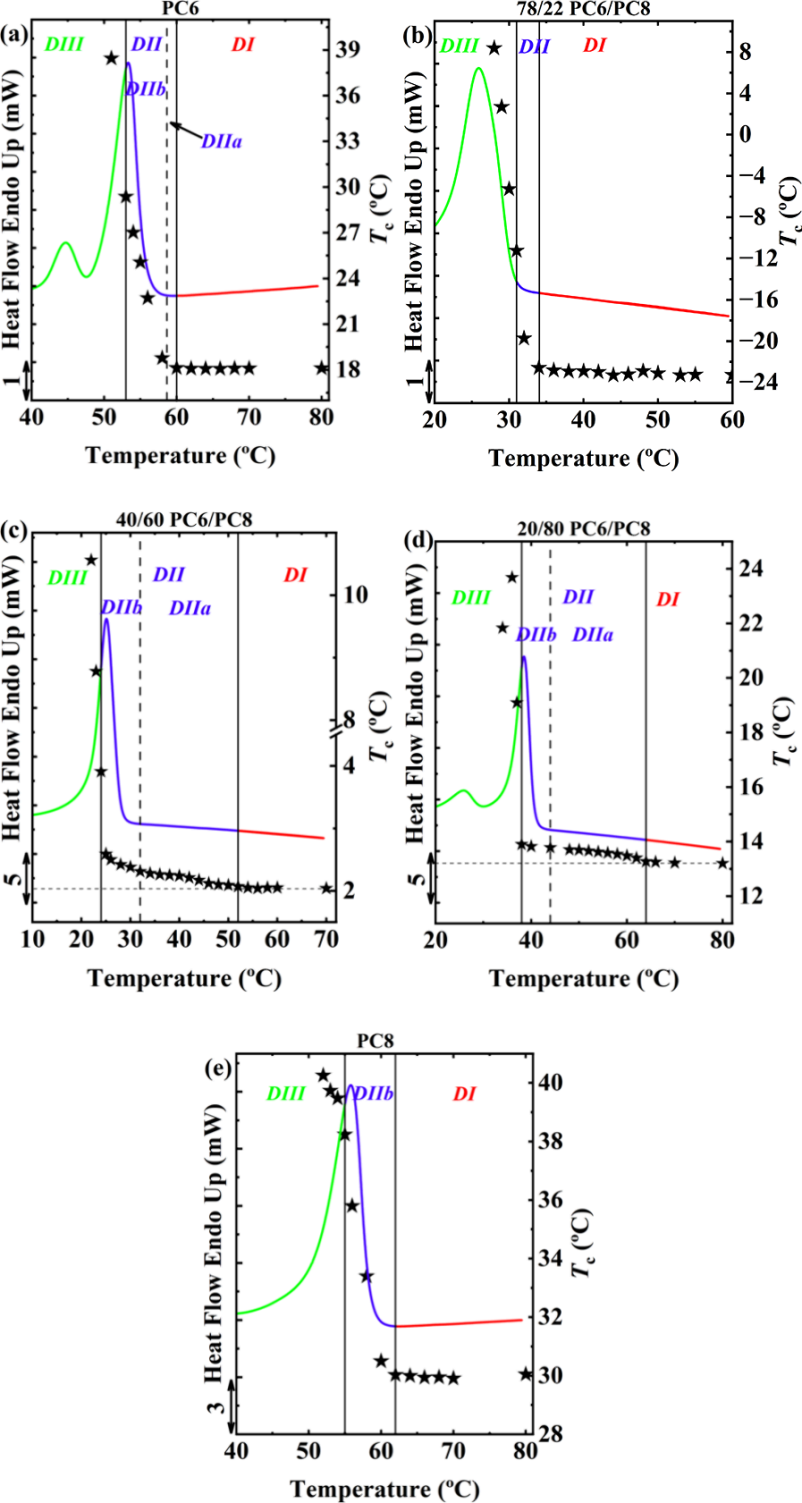
Crystallization temperature *T_c_* as a function of self-nucleation temperature *T_s_* superimposed on the standard DSC melting endotherm for different samples: (a) PC6, (b) 78/22 PC6/PC8, (c) 40/60 PC6/PC8, (d) 20/80 PC6/PC8, and (e) PC8. The vertical solid lines mark the boundaries between different *Domains*, while dashed lines indicate the end of the melting peak, further dividing *Domain II* into two sub-*Domains*: *Domain IIa* (*DIIa*) and *Domain IIb* (*DIIb*).

The width of *DIIa* is commonly used to quantify the strength of melt memory.^10^ As shown in Figures 3a and 3e, the reference homopolymers PC6 and PC8 exhibit very narrow *DIIa* regions, indicating weak melt memory. For the PC6-rich copolymer with a 78/22 composition, *DIIa* is absent, and only self-seeding in *DIIb* can be observed in Figure 3b, suggesting that incorporation of PC8 comonomers has eliminated the melt memory of PC6 crystals. This is in line with the isodimorphic crystallization of this composition and with previous findings regarding the influence of partial inclusion in isodimorphic copolyesters,^31^ which indicate that such comonomer inclusion represents a defect that interrupts intermolecular interactions, weakening melt memory.

However, the intermediate composition of 40/60 and the PC8-rich composition of 20/80 copolymers, where either the new γ phase or the γ phase coexists with the PC8-like phase, show significantly broader *DIIa* regions than the homopolymers, indicating a stronger melt memory. This unexpected increase in melt memory is likely related to intermolecular interactions within the γ phase, which will be explained in detail below.

To further investigate the impact of comonomer inclusion within the crystal lattice on the strength of melt memory, Figure 4 depicts the width of *DII*, including both the melt memory *Domain* (*DIIa*) and the self-seeding *Domain* (*DIIb*),^9,14,33^ as a function of PC8 content across all three copolymer series. In the case of PC6/PC8 system, the initial incorporation of PC8 comonomers leads to the complete disappearance of *DIIa* of the PC6 phase, while the width of *DIIb* remains nearly unchanged. This suggests that PC8 units act as defects within the PC6 lattice, as expected in a typical isodimorphic copolymer.^31^ Surprisingly, with a further increase in PC8 content, the melt memory of the copolymers not only re-emerges but is significantly amplified, far exceeding that of the parent components. This is evidenced by a dramatic broadening of *DIIa*, reaching a maximum width of 28 °C, which is even greater than that reported in polyamides and polyesters.^19,20,34,35^ Eventually, as PC8 becomes the dominant component and the PC6 content progressively decreases, the *DIIa* width narrows and ultimately vanishes in the PC8 homopolymer without any γ phase, indicating the disappearance of melt memory once again.

**Figure 4:**
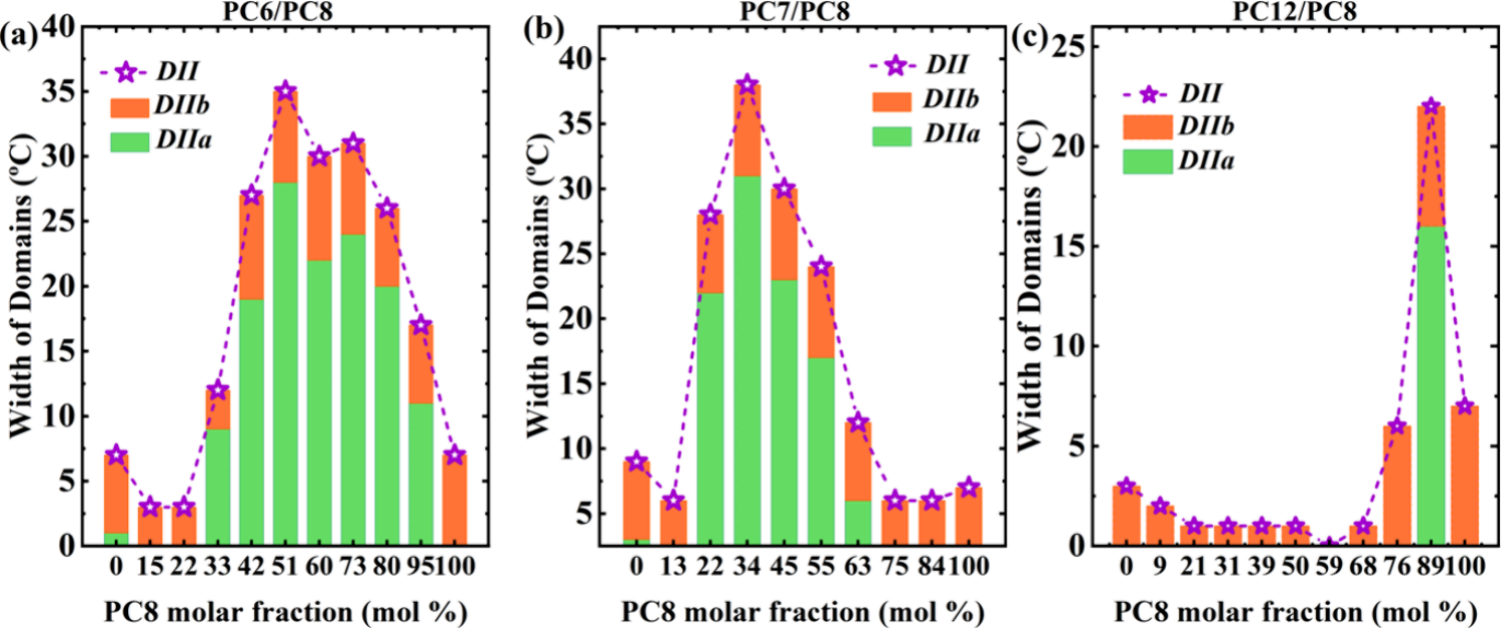
Widths of *DII*, *DIIa*, and *DIIb* as a function of PC8 content for (a) PC6/PC8, (b) PC7/PC8, and (c) PC12/PC8.

A similar anomalous enhancement of melt memory at intermediate compositions was observed in the PC7/PC8 copolymer system. Specifically, a sharp increase in *DIIa* width is detected within the 22–63 mol % PC8 compositions, indicating a pronounced melt memory. In contrast, the PC12/PC8 copolymers largely follow the typical behavior seen in isodimorphic PBS/PCL copolymers,^31^ where *DIIa* disappears, suggesting the absence of melt memory. An exception is found in the 11/89 PC12/PC8 sample, which displays an unusually broad *DIIa*, indicating strong melt memory at this specific composition. In this case, the formation of the γ phase and PC8-type phase explains such strong melt memory.

As shown in previous studies on copolyesters, the crystallization conditions, fast vs slow cooling, can influence the comonomer inclusion/exclusion balance.^36^ To account for this factor, we performed a structural characterization at selected *T* *_s_* following the SN protocol conducted in DSC experiments. Figure 5 shows representative WAXS patterns of PC6/PC8 collected during cooling after 5 min at *T*_s_, for compositions in which either (i) the parent phase coexists with the new γ phase, or (ii) only the γ phase forms under nonisothermal conditions. The WAXS patterns demonstrate that the new phase, identified by its reflections denoted Plane I and Plane II in Figure 5, or the coexistence of the γ phase with the parent component phase, can also develop under SN conditions, thereby preserving the mixed isodimorphic/isomorphic crystallization mode. More importantly, the data confirm that the changes observed in the DSC experiments depend on the crystalline phase formed, and thus on the dominant crystallization mode, isodimorphism or isomorphism. Further details on the structural characterization of these systems are provided in our previous wroks.^4,5^

**Figure 5:**
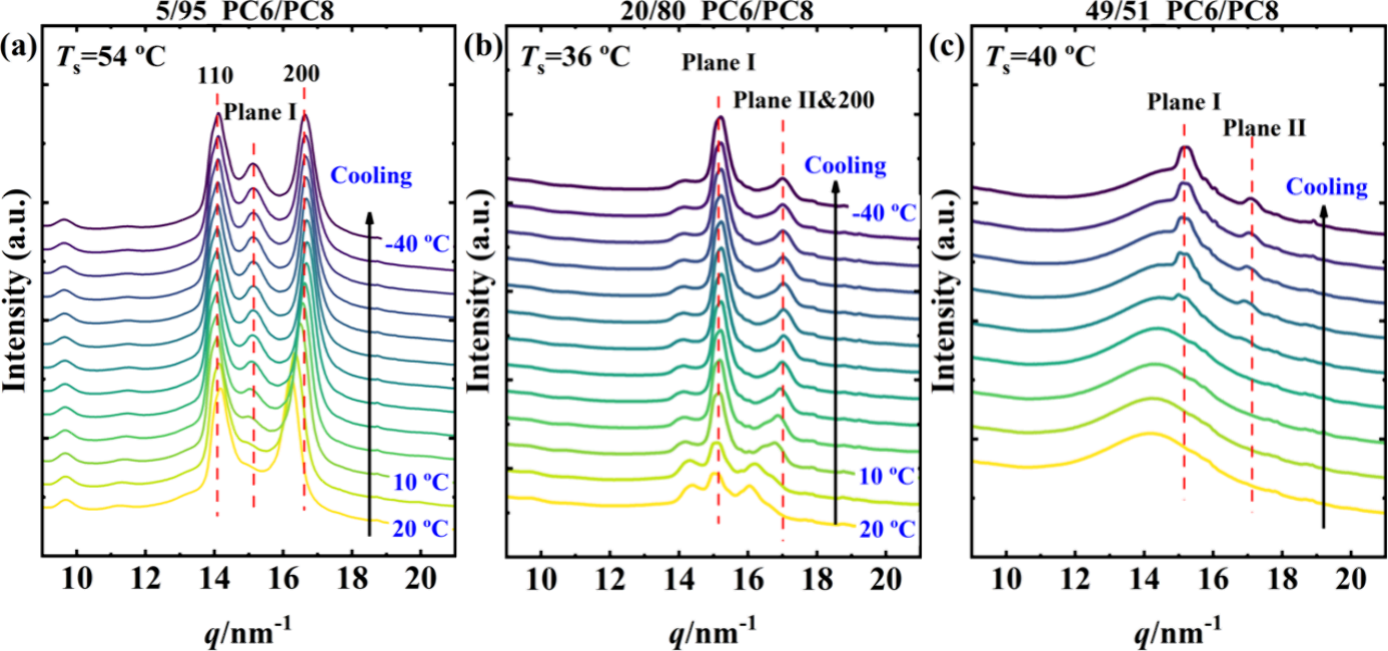
WAXS profiles obtained during the cooling process after self-nucleation at different *T*_s_ for representative copolymers of (a) 5/95, (b) 20/80, and (c) 49/51 PC6/PC8.

Considering above WAXS results conducted under SN conditions, it is clear that the width of *DIIa* depends on the crystalline structure, since those compositions with the broader *DIIa* correspond to either the compositions with the new γ phase or those where the γ phase and the phase of the parent components coexist. Thus, the stronger melt memory can be directly attributed to the formation of the γ phase in these systems. In this phase, comonomers are believed to share the same crystal lattice,^1,2,4^ representing a case of local isomorphism in otherwise isodimorphic copolymers. In the PC6/PC8 series, compositions such as 20/80 and 5/95, though outside the pure isomorphic window, still crystallize in the γ phase alongside the PC8 phase, which accounts for their broad *DIIa*. According to the WAXS results in Figure S5a, the 20/80 copolymer has a higher γ phase content than the 5/95 composition, correlating with its wider *DIIa.*

By comparing the melt memory among the different compositions and the dominant phase, it is clear that when the new γ phase is the dominant one, the melt memory is significantly higher. This indicates that the enhanced melt memory in these copolymers is primarily driven by γ phase formation, and its intensity is directly correlated with the γ phase content. In the case of PC12/PC8 copolymers, only the 11/89 copolymer exhibits a strong melt memory, as evidenced by the wider *DIIa* shown in Figure 4c, which means that the γ phase was generated in this copolymer.

### 3.3 Dielectric Spectroscopy Experiments

From the above results, it is clear that the new γ phase formation is behind the enhanced melt memory. Now, the question is what mechanism is responsible for such an enhancement? In olefin-based random copolymers, melt memory is predominantly attributed to the presence of a complex melt topology.^25,28,37–42^ Additionally, in copolyolefins in which a large amount of comonomer is excluded, the enhanced melt memory has been attributed to locally segregating long crystallizable sequences in the melt at proximity, selected by the initial crystallization process.^15,25^ However, in polymers containing functional groups such as polyamides, polyethers, and polycarbonates, the origin of melt memory is now widely attributed to intermolecular interactions between polar groups. These interactions stabilize self-nuclei in the melt and thus necessitate heating the polymer to temperatures well above the melting point to achieve an isotropic melt.^9,19,43,44^ Stronger intermolecular interactions typically result in a more pronounced melt memory. In isodimorphic copolymers, the partial inclusion of secondary comonomers into the crystal lattice of the dominant component tends to disrupt these interactions, thereby eliminating melt memory.^31^ However, this explanation does not apply to certain compositions, i.e., those in which a new phase is formed, for the PC8-based copolycarbonates studied in this work.

Here, for specific compositions, a mixed isodimorphic/isomorphic behavior has been demonstrated based on the formation of a new γ phase, in which total comonomer inclusion occurs.^4^ Therefore, in the γ phase, it is speculated that the dipole–dipole interactions between adjacent carbonyl (C = O) groups are not only preserved upon comonomer incorporation but may even be significantly strengthened. In fact, our previous work demonstrated this hypothesis using FT-IR experiments through systematic changes in the ν­(C = O) band position.^5^ To gain further understanding, we have performed complementary dielectric spectroscopy measurements here. Dielectric spectroscopy measurements were performed to confirm the existence of stronger intermolecular interactions in the γ phase. To optimize both time and signal quality for the following samples, a comparative experiment was carried out under both SN conditions (self-nucleated melt, *Domain IIa*) and nonisothermal (isotropic melt) conditions, as shown in Figure 6, yielding equivalent results.

**Figure 6:**
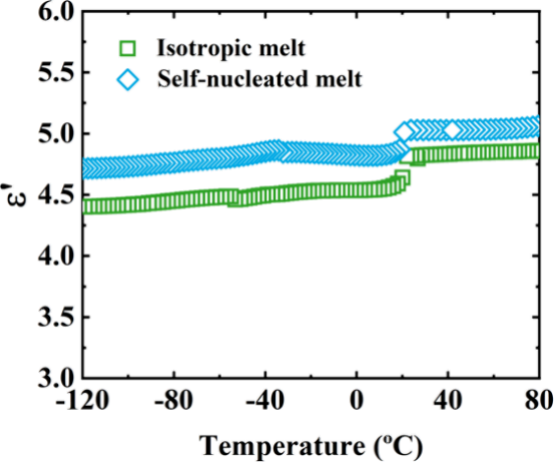
Dielectric constants ε*’* as a function of temperature for 49/51 PC6/PC8 copolymer after crystallization from isotropic melt and self-nucleated melt in *DIIa*.

Figure 6 shows the dielectric constant (ε*’*) as a function of temperature for the 49/51 PC6/PC8 composition, where the melt memory is most pronounced. Although ε*’* values are slightly higher under SN conditions, both crystallization protocols exhibit a sharp increase within the same temperature range (related to *T*_m_), consistent with the DSC results. Based on this equivalence, subsequent dielectric experiments were conducted using samples melted isotropically.

Figure 7 compares the ε*’* of representative PC6/PC8 copolymers under temperature-sweep (Figure 7a) and frequency-sweep (Figure 7b) modes. At a fixed frequency (Figure 7a) of 1000 Hz, ε*’* increases steadily with temperature and rises sharply between 30 and 60 °C, coinciding with copolymer melting. This behavior reflects enhanced chain mobility, which promotes dipole reorientation and relaxation polarization of polar groups,^45,46^ in complete agreement with the DSC results (Figure S5).

**Figure 7:**
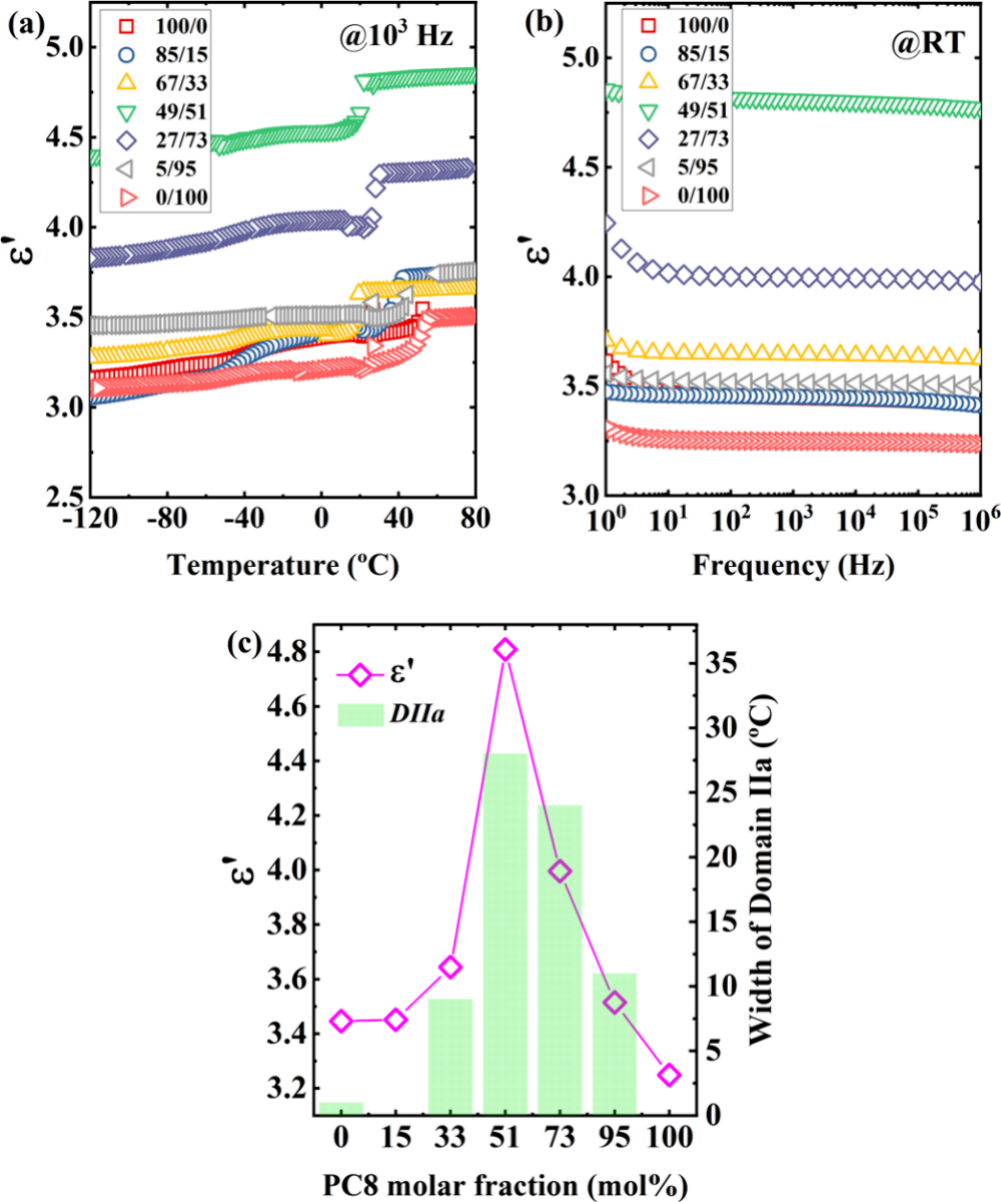
Dielectric constants ε*’* as a function of (a) temperature and (b) frequency for representative PC6/PC8 copolymers. In (c) ε*’* and the width of *Domain IIa* are compared as a function of comonomer content.

Isothermal frequency sweeps (Figure 7b) further highlight the role of copolymer composition. All copolymers display higher ε*’* than the homopolymers, with the 49/51 composition standing out as the most polar, exhibiting the strongest dipolar interactions across the frequency spectrum. The 27/73 and 67/33 copolymers, which crystallize in the pure γ phase, also display enhanced dipole–dipole interactions. Among the homopolymers, PC6 exhibits higher ε*’* than PC8, consistent with the dilution of dipole moments by PC8’s longer aliphatic chain. Interestingly, while small comonomer additions leave ε*’* nearly unchanged in the 85/15 copolymer, the 5/95 copolymer shows a slight increase relative to PC8, directly linked to γ phase formation.

Most strikingly, Figure 7c reveals a clear correlation between ε*’* (measured at room temperature and 1000 Hz) and the width of the melt memory *Domain* (*DIIa*) as a function of PC8 content. Both parameters exhibit the same compositional dependence, suggesting that the increased melt memory in the γ phase is strongly linked to stronger intermolecular dipolar interactions. These findings demonstrate that the pronounced melt memory observed in the isomorphic region is a distinctive feature of mixed isodimorphic/isomorphic crystallization.

## 4 Conclusions

In this work, we investigated the self-nucleation behavior and melt memory of three PC8-based copolycarbonates (PC6/PC8, PC7/PC8, and PC12/PC8), which crystallize through a mixed isodimorphic/isomorphic mode. A key finding is that melt memory is markedly enhanced when copolymers crystallize into the new γ phase, where total comonomer inclusion occurs. In contrast, compositions that crystallize isodimorphically, dominated by homopolymer-like crystals with only partial comonomer incorporation, lose their melt memory as expected, since comonomers act as lattice defects that disrupt intermolecular interactions. A direct correlation was established between the presence of the γ phase and the width of *Domain IIa*, a measure of melt memory strength. Dielectric spectroscopy measurements further support these findings, showing that γ phase crystals possess higher dielectric constants, consistent with stronger dipole–dipole interactions. This study demonstrates for the first time that an enhanced melt memory emerges as a new and distinctive feature of random copolymers crystallizing through a mixed isodimorphic/isomorphic mode. Unlike conventional isodimorphic random copolymers, where comonomers merely disrupt crystallinity, the γ phase introduces local isomorphism that enables full comonomer inclusion, preserving and even reinforcing intermolecular interactions. These findings provide new insights into the molecular origin of melt memory in polar random copolymers.

## Supplementary Material



## Notes

^1^H NMR and ^13^C NMR spectra of samples, thermal protocols, SN results, PLOM images, and FFT analysis process (PDF)
