# Supplementary material for: Understanding Enhanced Melt Memory in Poly(Octamethylene Carbonate)-Based Random Copolycarbonates with Mixed Isodimorphic/Isomorphic Crystallization
Source: Macromolecules. 2026 Jan 1;59(1):165–74. doi: 10.1021/acs.macromol.5c02607 (PMC12805643; doi:10.1021/acs.macromol.5c02607)
Supplement: Supplementary file 1 [file ma5c02607_si_001.pdf]

Understanding Enhanced Melt Memory in Poly(octamethylene carbonate)-Based Random Copolycarbonates with Mixed Isodimorphic/Isomorphic Crystallization

Yilong Liao[1,2], Ricardo A. Pérez-Camargo[3,1], Jon Maiz[4,5], Alejandro J. Müller[3,5,]*

[1] School of Materials Science and Engineering, and State Key Laboratory of High Performance Roll Materials and Composite Forming, Tianjin University, Tianjin 300072, P.R. China.

[2] State Key Laboratory of Advanced Polymer Materials, Sichuan University, Chengdu 610065, P.R. China.

[3] POLYMAT and Department of Polymers and Advanced Materials: Physics, Chemistry and Technology, Faculty of Chemistry, University of the Basque Country UPV/EHU, Donostia-San Sebastián 20018, Spain.

[4] Centro de Física de Materiales (CFM-MPC), CSIC-UPV/EHU, Paseo Manuel de Lardizabal 5, 20018, Donostia-San Sebastian, Spain.

[5] Ikerbasque, Basque Foundation for Science, Plaza Euskadi 5, 48009 Bilbao, Spain.

[1] *Corresponding authors: ricardoarpad.perez@ehu.eus; alejandrojesus.muller@ehu.es*

Section S1: Chemical Characterization for Random Copolycarbonates

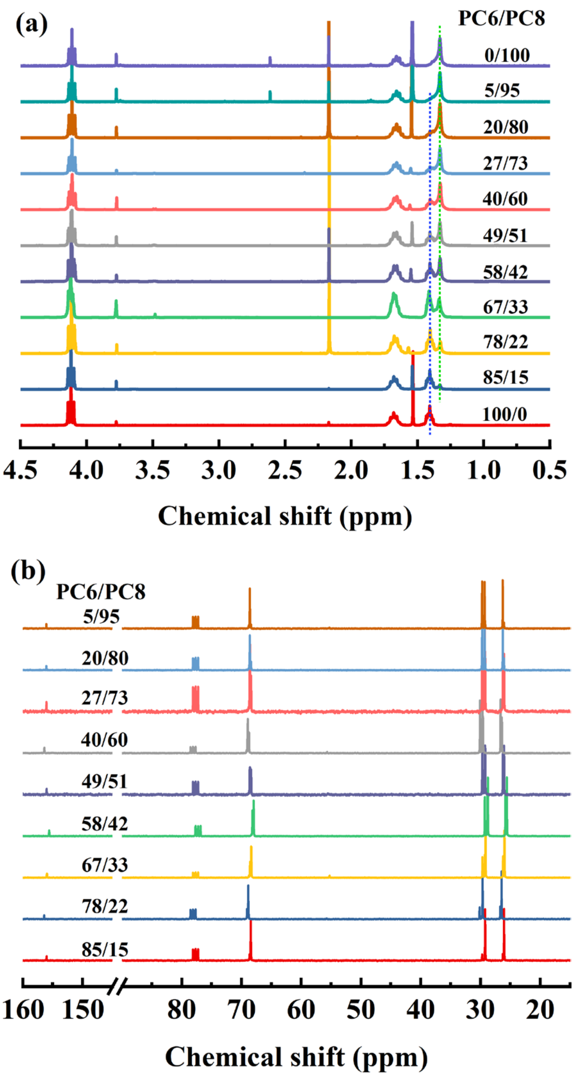

Figure S1. (a) ^{1}H NMR and (b) ^{13}C NMR spectra of PC6/PC8 copolymers in $CDCl_3$.

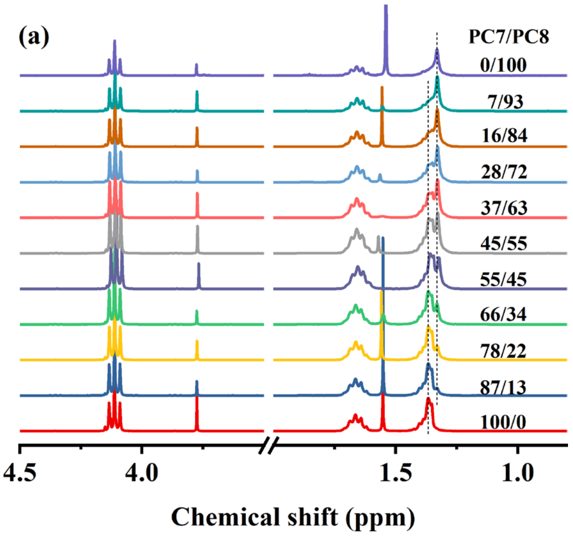

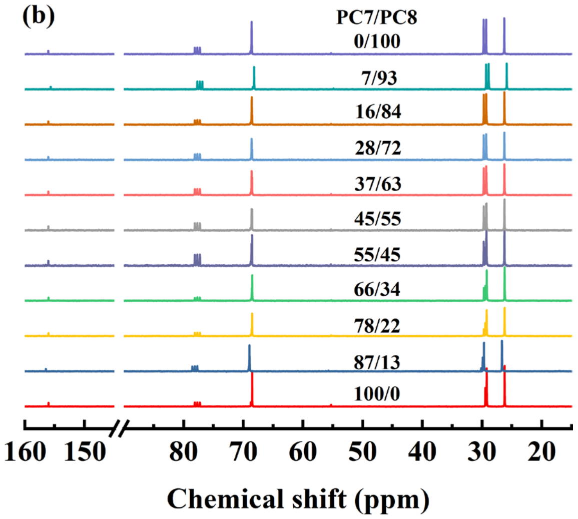

Figure S2. (a) ^{1}H NMR and (b) ^{13}C NMR spectra of PC7/PC8 copolymers in $CDCl_3$.

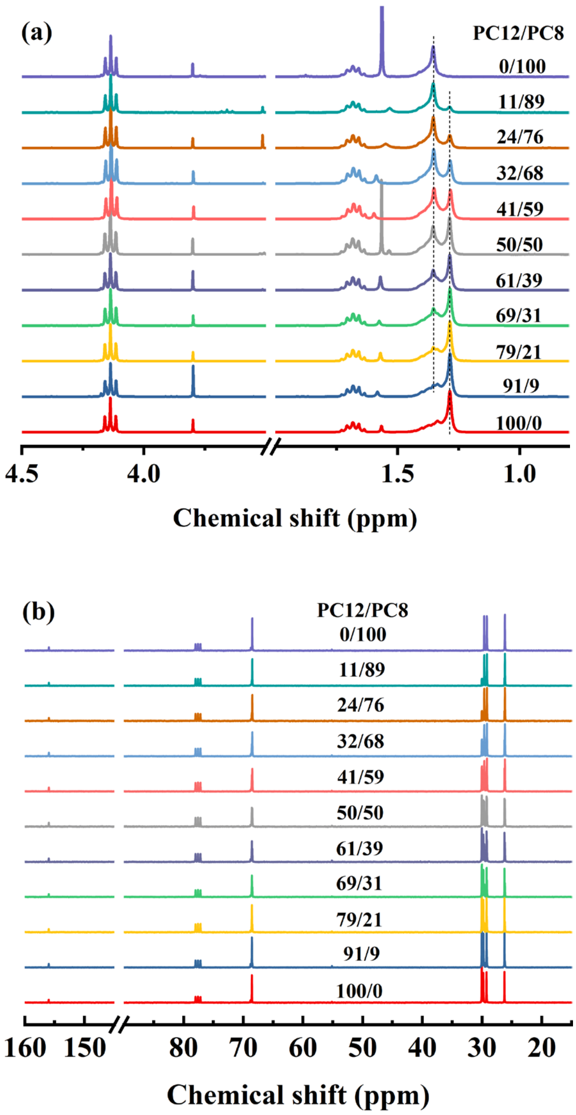

Figure S3. (a) ^{1}H NMR and (b) ^{13}C NMR spectra of PC12/PC8 copolymers in $CDCl_3$.

Table 3.1 Composition and Microstructure of PC6/PC8 Copolymers and Parent Homopolymers

PC6/PC8	[a] Composition (mol %)		[b] Dyad Content (mol %)			Sequence Length		[c] R	[c] M_w (kg/mol)	[c] $Đ$
	X_{PC6}	X_{PC8}	C6-C6	C6-C8 /C8-C6	C8-C8	N_{C6}	N_{C8}			
100/0	100.0	0.0	100.0	0.0	0.0	--	--	--	28	1.92
85/15	84.7	15.3	78.7	19.0	2.3	9.2	1.2	0.9	8	1.92
78/22	77.9	22.1	60.5	32.4	7.1	4.7	1.4	0.9	19	2.28
67/33	67.2	32.8	44.0	41.7	14.3	3.1	1.7	0.9	14	2.10
58/42	58.0	42.0	35.9	48.5	15.7	2.5	1.7	1.0	15	2.39
49/51	49.1	50.9	25.0	51.0	24.0	2.0	1.9	1.0	16	2.10
40/60	39.5	60.5	14.5	47.0	38.5	1.6	2.6	1.0	10	1.86
27/73	27.0	73.0	10.5	45.1	44.4	1.5	3.0	1.0	22	2.19
20/80	20.0	80.0	3.6	35.5	60.9	1.2	4.4	1.0	16	2.11
5/95	5.0	95.0	0.4	20.3	79.3	1.0	8.8	1.1	13	2.21
100/0	0.0	100.0	0.0	0.0	100.0	--	--	--	14	2.05

[a] Compositions of copolymers were calculated by ^{1}H NMR, and the copolymers were named x/y PC6/PC8, where the subscripts of x and y represent the molar percent (as integers) of PC6 and PC8, respectively. [b] The sequence distributions of C6-C6, C6-C8 or C8-C6, and C8-C8 dyads were calculated based on the intensity ratio of the signals

appearing around 67.8 ppm in the ^{13}C NMR spectra. The degree of randomness was determined from the average sequence lengths. ^{c} The weight-average molecular weight M_w and dispersity $Đ$ were determined through SEC.

Table S2. Composition and Microstructure of PC7/PC8 Copolymers and Parent Homopolymers

PC7/PC8	Composition (mol %)		Dyad Content (mol %)			Sequence Length		R	M_w (kg/mol)	$^{c}Đ$
	X_{PC7}	X_{PC8}	C7-C7	C7-C8 /C8-C7	C8-C8	N_{C7}	N_{C8}			
100/0	100.0	0.0	100.0	0.0	0.0	--	--	--	15	2.02
87/13	87.2	12.7	75.6	24.4	0.0	7.2	1.0	1.1	15	2.15
78/22	77.8	22.2	60.9	32.1	7.1	4.8	1.4	1.0	12	2.00
66/34	66.4	33.6	42.9	45.9	11.2	2.9	1.5	1.0	18	2.49
55/45	54.5	45.5	35.8	49.1	15.1	2.5	1.6	1.0	9	2.42
45/55	45.0	55.0	25.0	51.7	23.3	2.0	1.9	1.0	16	2.40
37/63	37.1	62.9	19.1	47.9	33.0	1.8	2.4	1.0	12	2.16
28/72	27.5	72.5	9.3	44.8	45.9	1.4	3.0	1.0	13	2.13
16/84	15.5	84.5	6.0	37.8	56.2	1.4	3.0	1.0	12	2.78
7/93	7.0	93.0	0.0	21.8	78.2	1.0	8.1	1.1	12	2.19
100/0	0.0	100.0	0.0	0.0	100.0	--	--	--	14	2.05

Table S3. Composition and Microstructure of PC12/PC8 Copolymers and Parent Homopolymers

PC12/PC8	[a] Composition (mol %)		[b] Dyad Content (mol %)			Sequence Length		[c]R	[c]M_w (kg/mol)	[c]$Đ$
	X_{PC12}	X_{PC8}	C12-C12	C12-C8 /C8-C12	C8-C8	N_{C12}	N_{C8}			
100/0	100.0	0.0	100.0	0.0	0.0	--	--	--	16	2.04
91/9	90.7	9.3	78.4	21.6	0.0	6.3	1.0	1.1	13	2.32
79/21	79.4	20.6	61.0	35.0	4.0	4.5	1.2	1.0	22	2.12
69/31	69.2	30.7	47.1	42.9	10.0	3.2	1.5	1.0	20	2.08
61/39	60.8	39.2	37.1	48.1	14.8	2.5	1.6	1.0	12	2.49
50/50	50.0	50.0	26.2	49.8	24.0	2.1	2.0	1.0	17	2.22
41/59	41.4	58.6	17.8	48.4	33.8	1.7	2.4	1.0	23	2.16
32/68	32.1	67.9	10.6	43.0	46.4	1.5	3.8	1.0	23	2.03
24/76	23.6	76.3	4.6	34.4	60.9	1.3	4.5	1.0	25	2.00
11/89	10.8	89.2	8.3	18.8	72.9	1.9	17.1	0.6	24	2.03
100/0	0.0	100.0	0.0	0.0	100.0	--	--	--	14	2.05

Section S2: Mixed Isomorphism/Isodimorphism in Random Copolycarbonates

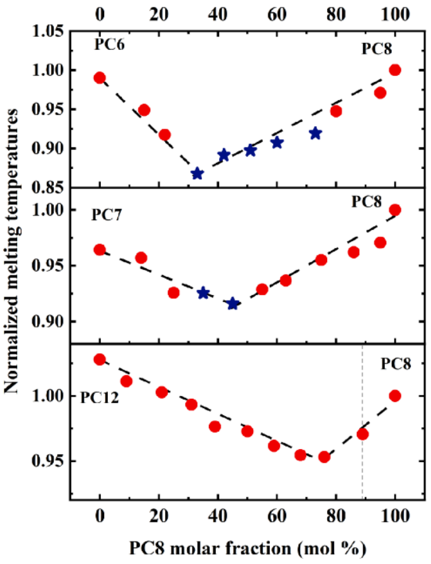

Figure S4. Normalized melting temperatures as a function of PC8 molar fraction for PC6/PC8, PC7/PC8, and PC12/PC8 random copolymers. The star points represent the melting of pure γ phase.[1]

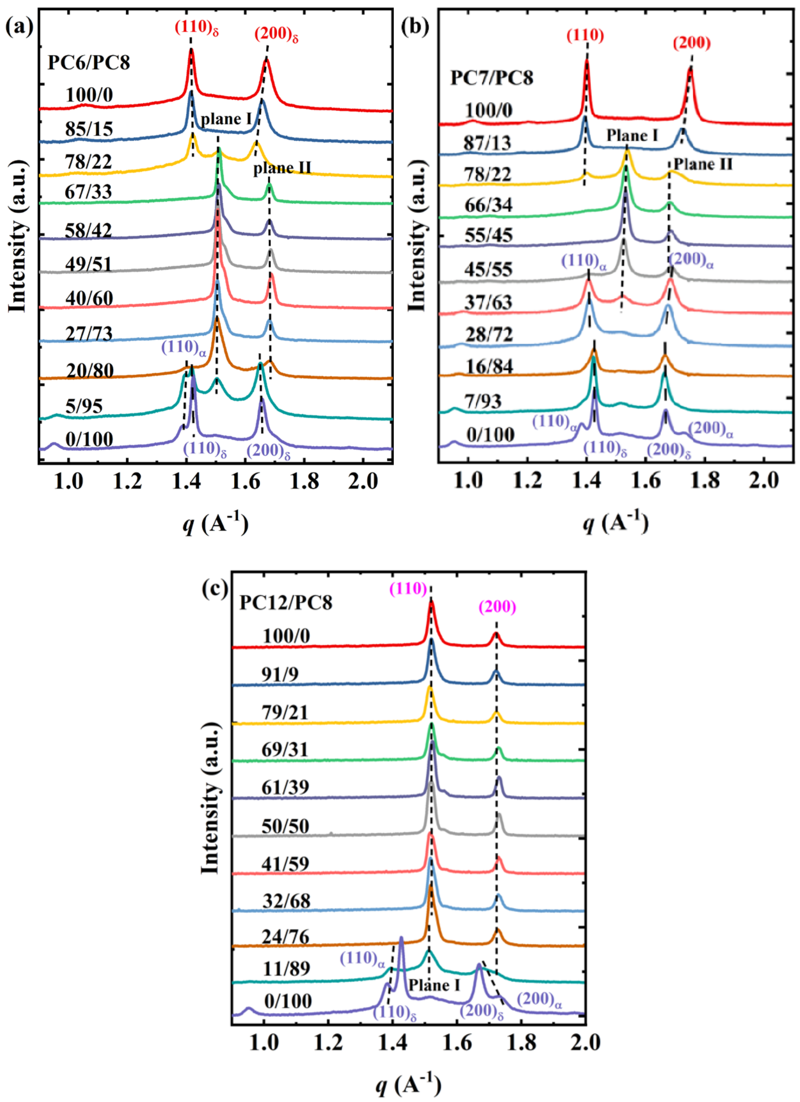

Figure S5. WAXS patterns of (a) PC6/PC8, (b) PC7/PC8, and (c) PC12/PC8 copolymers after crystallization from isotropic melt.[1, 2]

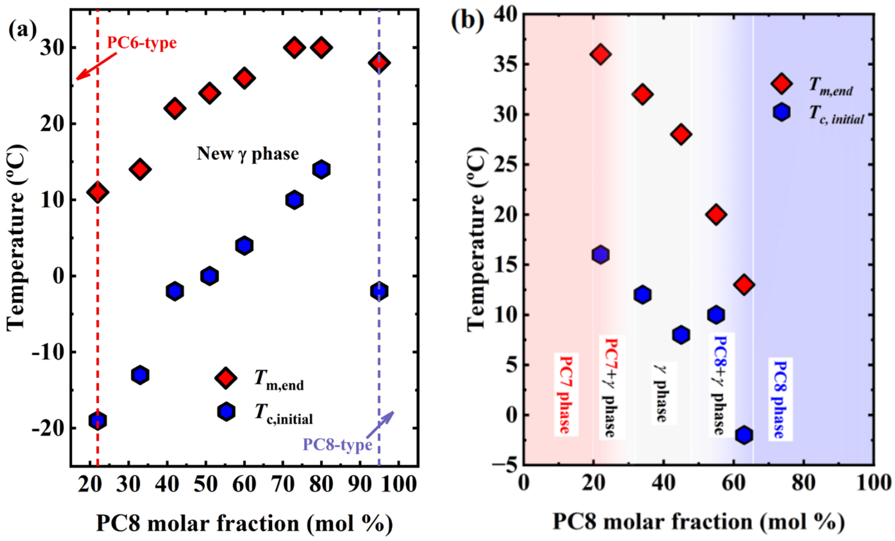

Figure S6. Final melting temperature ($T_{m,end}$) and initial crystallization ($T_{c,initial}$) temperature of the γ phase as a functionof PC8 molar fraction for (a) PC6/PC8 and (b) PC7/PC8 copolymers. The temperatures were obtained from the appearance and disappearence of the characteristic diffraction peak corresponding to the γ phase in the in-situ WAXS patterns.[1, 2]

Section S3: Self-nucleation Experiments.

The typical thermal program of self-nucleation was proposed by Fillon et al.[3] and extensively used and reviewed by Müller et al.[4, 5] It consists of five steps at the scanning rate of 20 °C/min:

(1) The sample is first heated to a sufficiently high temperature, typically 30 °C above its melting point, and held there for 3 minutes to erase any thermal history and achieve an isotropic melt. This process effectively destroys any thermally sensitive nuclei, leaving only temperature-resistant heterogeneous nuclei of unknown origin, such as impurities, catalysts, or excipients in industrial samples.

(2) The isotropic melt is cooled at 20 °C/min to -40 °C, which is then maintained for 1 minute to achieve a standard semi-crystalline state. The exothermic peak

observed during cooling is defined as the "standard" crystallization temperature (T_c). This standard state can be reproduced in any subsequent self-nucleation (SN) experiment, provided the cooling rate and temperature parameters are consistent.

(3) The standard crystals are heated up to a temperature of self-nucleation, denoted as T_s, and then the sample is held at T_s for 5 min.

(4) A subsequent cooling scan from T_s to the minimum temperature chosen in step 2 could display the change in crystallization kinetics affected by the SN treatment.

(5) Finally, the sample was reheated to completely melt it, as described in step 1, and the melting behavior was recorded. In general, the difference in melt endotherms between *Domain I* and *Domain II* is negligible. However, the appearance of a new higher melting peak in addition to the main one is a sign of *Domain III*, as the annealed crystals have thicker lamellae and melt at a higher temperature.

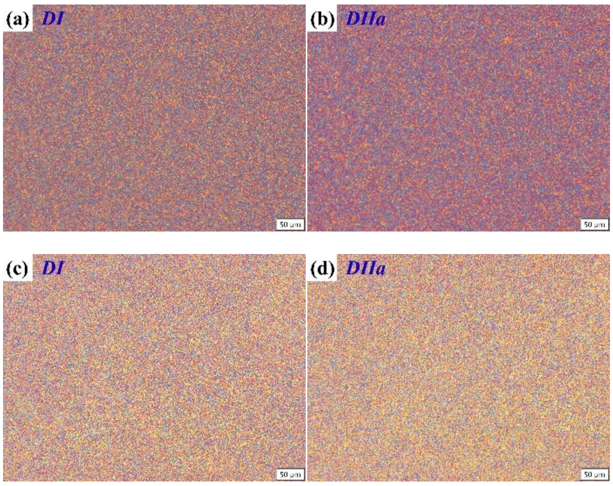

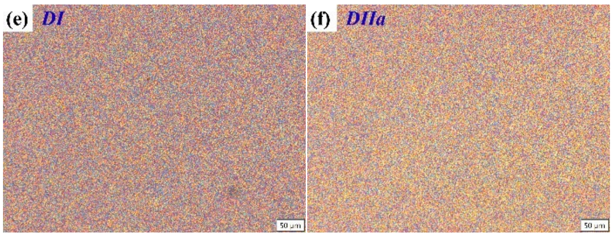

Figure S7. POM images captured at -40 °C after crystallization from different T_s for selected sample in PC6/PC8 system: (a, b) 58/42, (c, d) 40/60, and (e, f) 27/73.

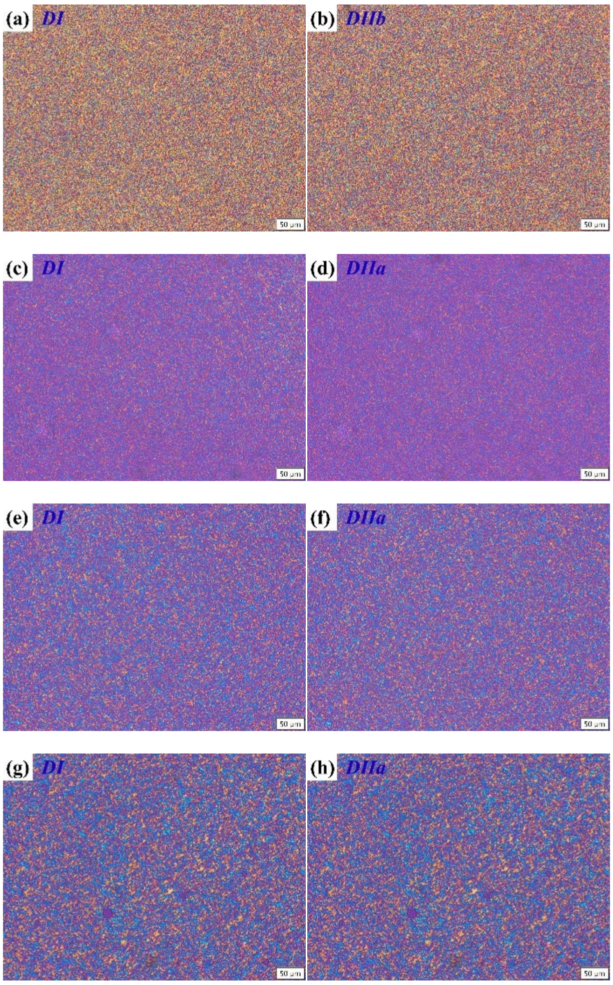

Figure S8. POM images captured at -40 °C after crystallization from different T_s for selected sample in PC7/PC8 system: (a, b) 78/22, (c, d) 66/34, (e, f) 55/45, and (g, h) 45/55.

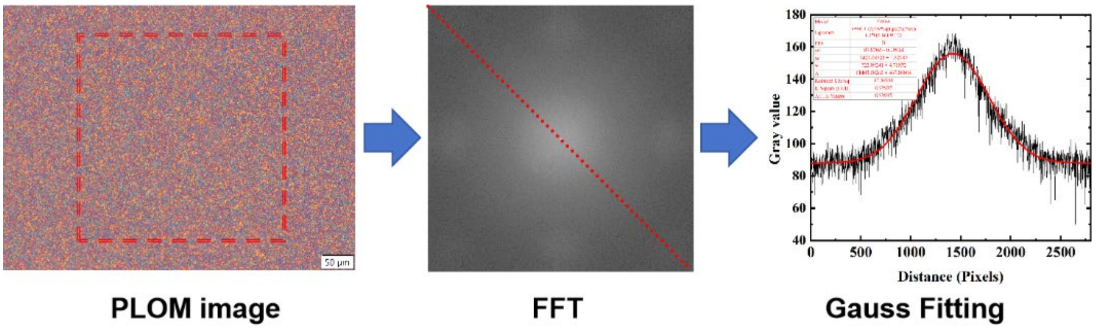

Figure S9. Example of Fast Fourier Transform analysis process of PLOM image and half-peak width calculation from Gauss fitting.

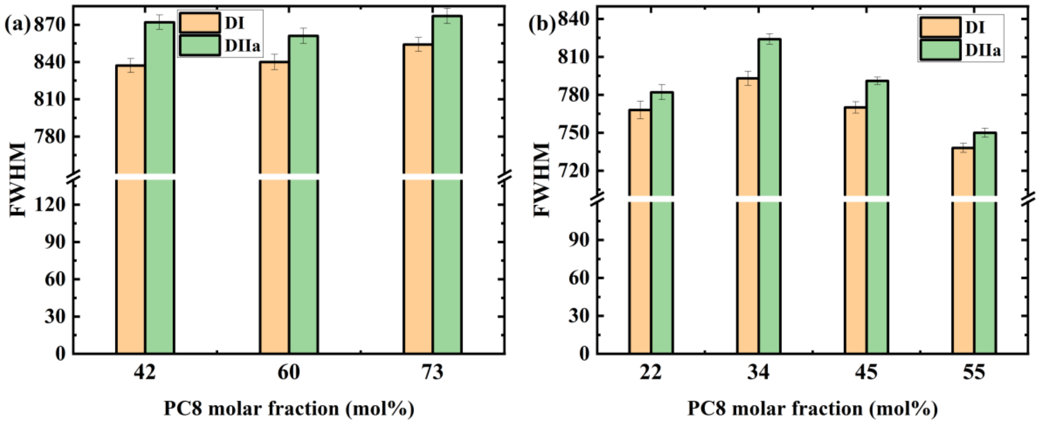

Figure S10. FWHM values obtained from Gauss fitting after FFT process on the PLOM images for representative copolymers in (a) PC6/PC8 and (b) PC7/PC8 system.

Note that for the materials in this study, the increase in T_c with decreasing T_s is quite minimal. This might be explained by considering that acceleration of crystallization kinetics in melt memory domain fundamentally relies on an increase in the density of self-nuclei. However, these copolymers intrinsically possess a high nucleation density, which may limit the extent to which additional self-nuclei can be generated during the self-nucleation. As a result, the enhancement in crystallization kinetics after self-nucleation is marginal. Figures S8-S9 captures the spherulitic morphologies of various copolymer system crystallized from isotropic melts (T_s

in *DI*) and self-nucleated melt (T_s in *DIIa*). These images clearly reveal that all copolymers crystallize into highly dense spherulitic structures.

To qualitatively evaluate changes in nucleation density before and after self-nucleation, Fast Fourier Transform (FFT) analysis was performed on the PLOM images.[6] As shown in Figure S11, a fixed-size region of each PLOM image was selected and transformed to FFT image using ImageJ software. Grayscale values were extracted along the diagonal of the FFT image to generate an intensity profile (gray value vs. distance), and Gaussian fitting was applied to determine the full width at half maximum (FWHM). A larger FWHM value corresponds to smaller average spherulite size and, by extension, a higher nucleation density. Figure 4 shows that the FWHM values derived from *DIIa* are consistently higher than those from *DI*, indicating smaller spherulite sizes. This might be attributed to the formation of self-nuclei in *DIIa*, which plausibly increases the nucleation density. However, the observed magnitude of increase in FWHM is rather limited, suggesting that the enhanced density is minimal, in agreement with the negligible changes observed in T_c.

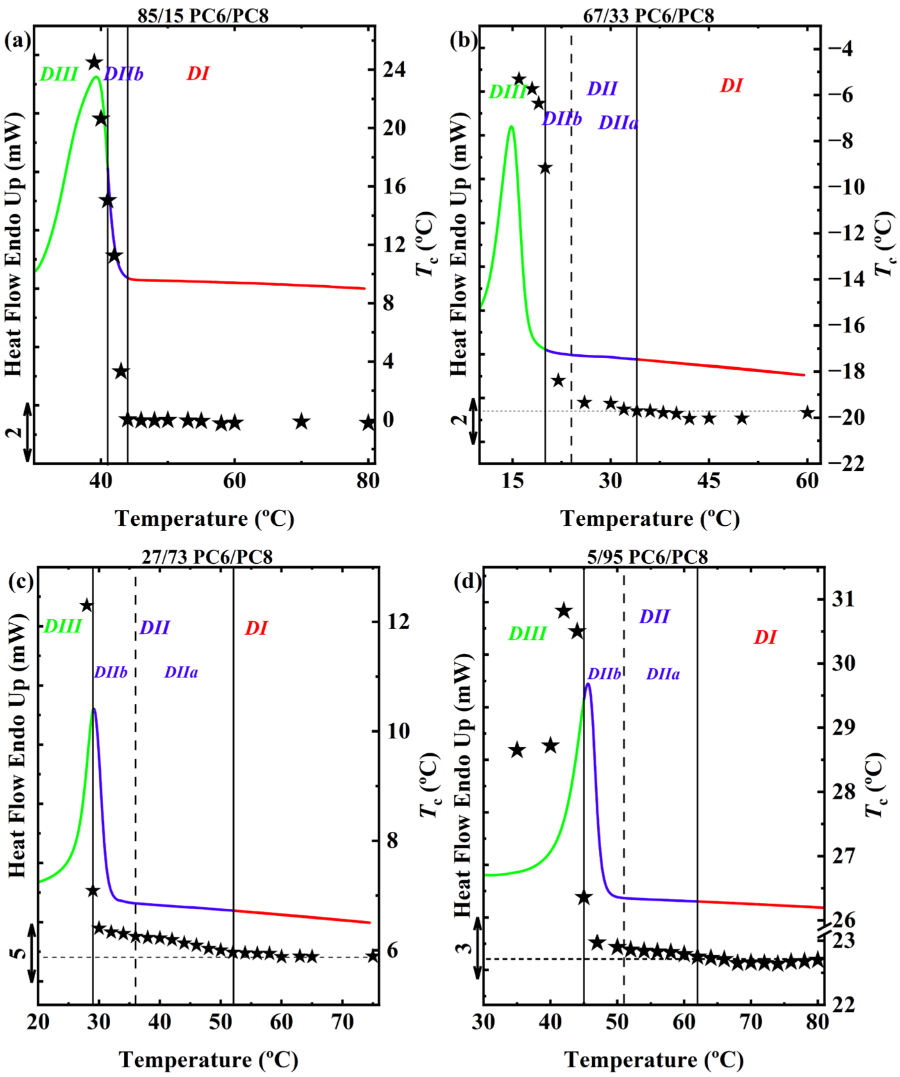

Figure S11. Crystallization temperature T_c as a function of self-nucleation temperature T_s, superimposed on the standard DSC melting endotherm for selected PC6/PC8 copolymers: (a) 85/15, (b) 67/33, (c) 27/73, and (d) 5/95.

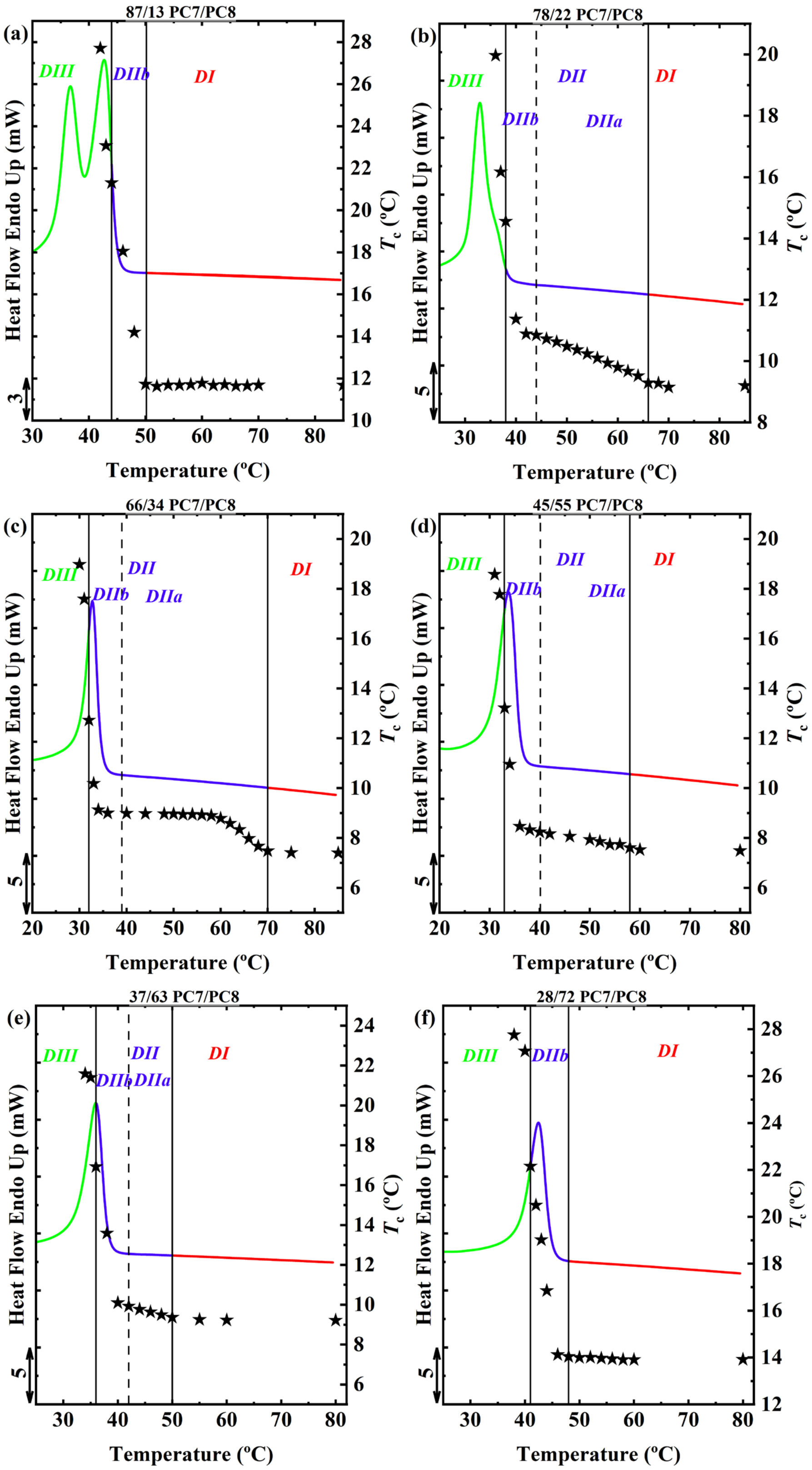
(a)
87/13 PC7/PC8
DIII
DIIb
DI
Heat Flow Endo Up (mW)
T_c (ºC)
Temperature (ºC)
(b)
78/22 PC7/PC8
DIII
DIIb
DII
DIIa
DI
Heat Flow Endo Up (mW)
T_c (ºC)
Temperature (ºC)
(c)
66/34 PC7/PC8
DIII
DIIb
DII
DIIa
DI
Heat Flow Endo Up (mW)
T_c (ºC)
Temperature (ºC)
(d)
45/55 PC7/PC8
DIII
DIIb
DII
DIIa
DI
Heat Flow Endo Up (mW)
T_c (ºC)
Temperature (ºC)
(e)
37/63 PC7/PC8
DIII
DIIb
DII
DIIa
DI
Heat Flow Endo Up (mW)
T_c (ºC)
Temperature (ºC)
(f)
28/72 PC7/PC8
DIII
DIIb
DI
Heat Flow Endo Up (mW)
T_c (ºC)
Temperature (ºC)

Figure S12. Crystallization temperature T_c as a function of self-nucleation temperature T_s, superimposed on the standard DSC melting endotherm for selected PC7/PC8 copolymers: (a) 87/13, (b) 78/22, (c) 66/34, (d) 45/55, (e) 37/63, and (f) 28/72.

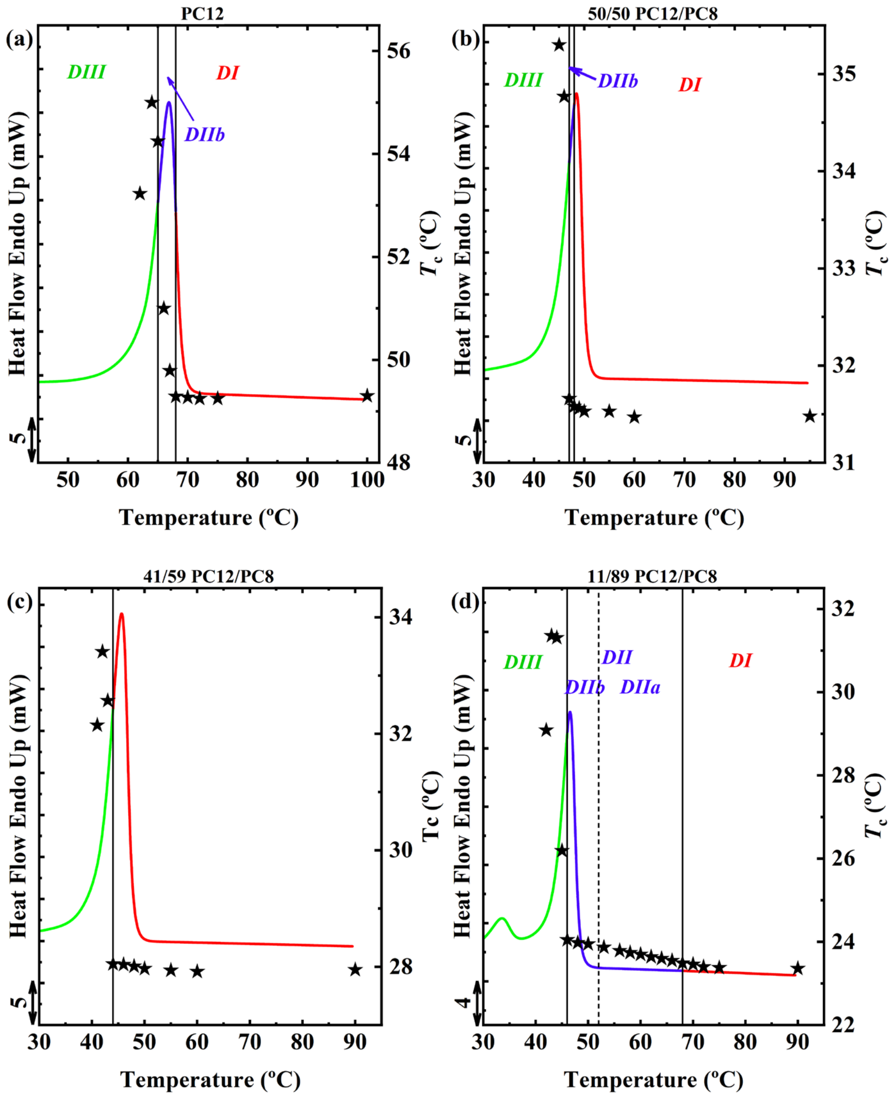

Figure S13. Crystallization temperature T_c as a function of self-nucleation temperature T_s, superimposed on the standard DSC melting endotherm for (a) PC12 and selected PC12/PC8 copolymers: (b) 50/50, (c) 41/59, and (d) 11/89.

Section S4: Determination of equilibrium melting temperatures

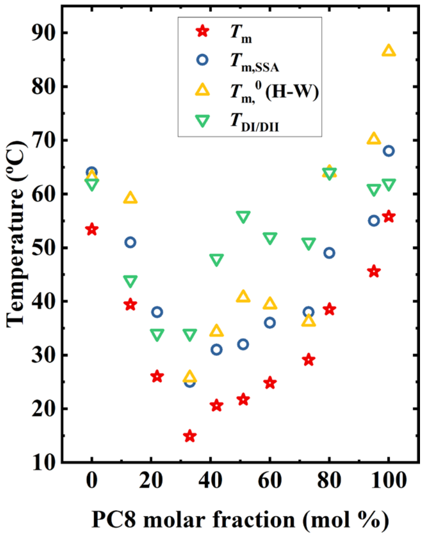

Figure S14. Melting temperature, the highest melting temperatures after SSA treatment, equilibrium melting temperature determined by Hoffman-Weeks extrapolation, and the boundary temperature between *Domain I* and *Domain II* as a function of PC8 content in PC6/PC8 system.

The equilibrium melting temperatures T_m^0 is the melting point of lamellar crystals with infinite thickness and negligible surface effects on melting, which represents the first order of a hypothetical macroscopic perfect crystals. The Hoffman-Weeks (H-W) extrapolation method is commonly used to estimate the T_m^0 of polymers, but it is not always reliable. In Figure S14, a comparison between the measured melting temperatures and the T_m^0 values estimated by the H-W method reveals a significant discrepancy in their trend. In contrast, the highest melting temperatures ($T_{m,SSA}$) obtained after successive self-nucleation and annealing (SSA) treatments show a much closer correlation with the observed melting temperature trends. Arandia et al.[7] reported that the equilibrium melting temperature can be approximately estimated by adding a constant value to the $T_{m,SSA}$. This method allows for effective comparison within the same polymer system. Therefore, in this case, we chose to estimate the equilibrium melting temperature of each sample by adding a fixed constant of 20 °C to the $T_{m,SSA}$ value.

References

1. Liao, Y.; Pérez-Camargo, R. A.; Ma, T.; Maiz, J.; Martínez de Ilarduya, A.; Sardon, H.; Liu, G.; Wang, D.; Müller, A. J., Mixed Isodimorphic/Isomorphic Crystallization in Aliphatic Random Copolycarbonates. *Macromolecules* **2024,** *57* (21), 10227-10239.
2. Liao, Y.; Pérez-Camargo, R. A.; Sardon, H.; Martínez de Ilarduya, A.; Hu, W.; Liu, G.; Wang, D.; Müller, A. J., Challenging Isodimorphism Concepts: Formation of Three Crystalline Phases in Poly(hexamethylene-ran-octamethylene carbonate) Copolymers. *Macromolecules* **2023,** *56* (20), 8199-8213.
3. Fillon, B.; Wittmann, J. C.; Lotz, B.; Thierry, A., Self -Nucleation and Recrystallization of lsotactic Polypropylene (α Phase) Investigated by Differential Scanning Calorimetry. *J. Polym. Sci., Part B: Polym. Phys.* **1993,** *31* (10), 1383-1393.
4. Michell, R. M.; Mugica, A.; Zubitur, M.; Müller, A. J., Self-Nucleation of Crystalline Phases Within Homopolymers, Polymer Blends, Copolymers, and Nanocomposites. *Adv. Polym. Sci.* **2017,** *276*, 215-256.
5. Sangroniz, L.; Cavallo, D.; Müller, A. J., Self-Nucleation Effects on Polymer Crystallization. *Macromolecules* **2020,** *53* (12), 4581-4604.
6. Li, Y.; Wang, S.; Wu, T.; Meng, X.; Ye, H.-M., Self-Nucleation Ability and Intermolecular Interactions Mechanism in Fluoropolyolefins. *ACS Appl. Polym. Mater.* **2024,** *6* (12), 7077-7087.
7. Arandia, I.; Zaldua, N.; Maiz, J.; Pérez-Camargo, R. A.; Mugica, A.; Zubitur, M.; Mincheva, R.; Dubois, P.; Müller, A. J., Tailoring the isothermal crystallization kinetics of isodimorphic poly (butylene succinate-ran-butylene azelate) random copolymers by changing composition. *Polymer* **2019,** *183*, 121863.
